# Accuracy of i-Scan for Optical Diagnosis of Colonic Polyps: A Meta-Analysis

**DOI:** 10.1371/journal.pone.0126237

**Published:** 2015-05-15

**Authors:** Chuan-Guo Guo, Rui Ji, Yan-Qing Li

**Affiliations:** Department of Gastroenterology, Qilu Hospital, Shandong University, Jinan, Shandong Province, China; University Hospital Llandough, UNITED KINGDOM

## Abstract

**Background:**

i-Scan is a novel virtual chromoendoscopy system designed to enhance surface and vascular patterns to improve optical diagnostic performance. Numerous prospective studies have been done to evaluate the accuracy of i-Scan in differentiating colonic neoplasms from non-neoplasms. i-Scan could be an effective endoscopic technique for optical diagnosis of colonic polyps.

**Objective:**

Our aim of this study was to perform a meta-analysis of published data to establish the diagnostic accuracy of i-Scan for optical diagnosis of colonic polyps.

**Methods:**

We searched PubMed, Medline, Elsevier ScienceDirect and Cochrane Library databases. We used a bivariate meta-analysis following a random effects model to summarize the data and plotted hierarchical summary receiver-operating characteristic (HSROC) curves. The area under the HSROC curve (AUC) serves as an indicator of the diagnostic accuracy.

**Results:**

The meta-analysis included a total of 925 patients and 2312 polyps. For the overall studies, the area under the HSROC curve was 0.96. The summary sensitivity was 90.4% (95%CI 85%-94.1%) and specificity was 90.9% (95%CI 84.3%-94.9%). In 11 studies predicting polyps histology in real-time, the summary sensitivity and specificity was 91.5% (95%CI 85.7%-95.1%) and 92.1% (95%CI 84.5%-96.1%), respectively, with the AUC of 0.97. For three different diagnostic criteria (Kudo, NICE, others), the sensitivity was 86.3%, 93.0%, 85.0%, respectively and specificity was 84.8%, 94.4%, 91.8%, respectively.

**Conclusions:**

Endoscopic diagnosis with i-Scan has accurate optical diagnostic performance to differentiate neoplastic from non-neoplastic polyps with an area under the HSROC curve exceeding 0.90. Both the sensitivity and specificity for diagnosing colonic polyps are over 90%.

## Introduction

Colorectal carcinoma (CRC) is a major public health burden worldwide. Colonoscopy has been widely accepted as the preferred modality for the early detection of CRC. Polypectomy, especially removal of adenomas, could disrupt the polyp-cancer sequence to reduce the incidence and mortality of CRC [1, 2]. However, about 90% of all colonic polyps are smaller than 1 cm in diameter and 80% are diminutive colon polyps (≤ 5 mm), which rarely have malignant potential, and more colonic polyps are identified during colonoscopy as the image resolution of instruments improved [3]. As a consequence, the cost of histological assessment of colorectal polyps has risen, accounting for 30%–50% of all surgical pathology costs [3]. If a sufficiently accurate real-time optical diagnosis of polyps could be made, this may allow the application of a “resect and discard” strategy for neoplastic diminutive colon polyps, and the endoscopists to leave diminutive rectosigmoid hyperplastic polyps in situ [4–6] which have negligible malignant potential [7], to reduce pathology costs.

The clinical application of the “resect and discard” strategy depends to a great extent on the accuracy of endoscopic optical diagnosis in real-time. To improve the accuracy of optical diagnosis of colon polyps, dye-based chromoendoscopy [8, 9], digital image-enhanced endoscopy such as narrow-band imaging (NBI, Olympus, Japan) [10–14], fujinon intelligent color enhancement (FICE, Fujinon, Japan) [15, 16], and image-enhanced endoscopy (i-Scan, Pentax,Japan), have been used in clinical practice. Among the image-enhancing techniques, i-Scan is a novel virtual chromoendoscopy system designed to enhance surface and vascular patterns to improve optical diagnostic performance in vivo [17].

Over the past few years, numerous prospective studies have been done utilizing i-Scan in real-time or post hoc (static images assessment) with different diagnostic criteria to evaluate the accuracy of i-Scan in differentiating colonic neoplasms from non-neoplasms with histology as the reference standard. Our aim of this study was to perform a meta-analysis of published data to establish the overall diagnostic accuracy (sensitivity and specificity) of i-Scan for optical diagnosis of colonic polyps especially in real-time.

## Materials and Methods

### Search strategy and study selection

Our meta-analysis was done in accordance with the Preferred Reporting Items for Systematic reviews and Meta-Analyses (PRISMA) guidelines (S1 Table.) [18]. We systematically searched the PubMed, Medline, Elsevier ScienceDirect and Cochrane Library databases for all articles associated with i-Scan and colonic polyps published until October 2014. Studies in PubMed were identified with the term i-Scan combined with the MeSH terms colonoscopy, colonic neoplasms or colonic polyps or words beginning with colorect, colon imag or colonoscop. We searched Elsevier ScienceDirect with the terms colon neoplasms or colon polyps and i-Scan with the topics restricted to “colorectal cancer, gastrointestinal, adeno carcinoma, rectal cancer”. Studies in Medline were identified with the terms colon polyps, colon neoplasms and i-Scan. We also searched the Cochrane Library for any systematic review that was relevant to our meta-analysis. Following the initial search, suitable articles on the basis of the titles and abstracts were identified, and then a detailed full text assessment of potentially relevant studies was performed. The reference lists of the relevant articles were checked to avoid missing related studies. Finally, we reviewed the identified studies to assess whether they were eligible according to the inclusion and exclusion criteria. Disagreements between investigators were resolved through discussion.

### Inclusion and exclusion criteria

The inclusion criteria were as follows:

1. Studies that used i-Scan prospectively evaluated patients undergoing colonoscopy for screening or surveillance;
2. Diagnostic clinical studies that evaluated the accuracy of i-Scan to make a prediction of polyps histology (neoplastic or non-neoplastic);
3. Studies that compared i-Scan with histology as the reference standard;
4. Studies with available data for constructing 2×2 tables with true positive (TP), false positive (FP), false negative (FN) and true negative (TN); and
5. Studies that were published in English language.

The exclusion criteria were as follows:

1. Studies without histology as the reference standard;
2. Studies without complete data for constructing 2×2 tables with true positive (TP), false positive (FP), false negative (FN) and true negative (TN);
3. Studies that overlapped the studies selected;
4. Studies that included patients with inflammatory bowel disease, familial polyposis syndromes, or colorectal cancer; and
5. Studies primarily designed as a retrospective study, review without original data, or meta-analysis.

### Data extraction

Two reviewers independently extracted data by using a standardized form designed by our group. If there was inconsistency, the original papers were retrieved and disagreements were resolved by discussion. The data of true positive (TP), false positive (FP), false negative (FN) and true negative (TN) were extracted with the histology as gold standard. We constructed 2×2 tables to record the number of polyps identified as TP (neoplastic polyps predicted to be neoplastic endoscopically), FP (non-neoplastic polyps predicted to be neoplastic endoscopically), FN (neoplastic polyps predicted to be non-neoplastic endoscopically), and TN (non-neoplastic polyps predicted to be non-neoplastic endoscopically). In addition, the following data were extracted for each study, if available, first author, publication year, country or area, type of study, number of patients enrolled, patients age, sex ratio, number of polyps, size of polyps, diagnostic criteria, histological reference standard, number of endoscopists, mode of i-Scan and endoscope used. Diagnostic criteria was classified into Kudo pit pattern classification [19, 20] or modified Kudo pit pattern classification (Kudo for short), the Narrow Band Imaging International Colorectal Endoscopic (NICE) classification [21, 22] and other criteria.

### Study quality assessment

Two reviewers independently assessed the quality and potential for bias of all studies by using the revised Quality Assessment of Diagnostic Accuracy Studies (QUADAS- 2) tool [23]. There are 4 phases of the QUADAS-2 tool: summarize the review question, produce review-specific guidance, construct a flow diagram, and judge bias and applicability. This tool comprised 4 domains to judge bias and applicability of the studies: patient selection, index test, reference standard, and flow and timing. Each domain was assessed in terms of risk of bias with signaling questions to help us judge risk of bias. The first 3 domains also had parts to assess in terms of concerns regarding applicability. A study would have an overall judgment of “low risk of bias” or “low concern regarding applicability”, if it was judged as “low” on all domains. In contrast, it would be judged as “risk of bias” or having “concerns regarding applicability”, if it was judged “high” or “unclear” in 1 or more domains.

### Statistical methods

A bivariate meta-analysis following a random effects model was used to calculate summary estimates of sensitivity and specificity and to plot a hierarchical summary receiver-operating characteristic (HSROC) curve [24, 25]. Positive likelihood ratio and negative likelihood ratio were calculated with the same model. We also calculated 95% confidence intervals (CI) for the summary estimates and likelihood ratios. All studies are presented as a circle and plotted with the HSROC curve. The summary point is represented by a dot which was surrounded by a 95% confidence region. The area under the HSROC curve was calculated.

The heterogeneity of the included studies was also measured. To find the source of heterogeneity, we performed subgroup analyses to assess the effect of assessment methods (histological prediction of colon polyps in real-time or not), diagnostic criteria (Kudo, NICE and others), polyps size (polyps ≤ 10 mm or polyps ≤ 5 mm), published type (full-text or abstracts), polyps number (< 200 and ≥ 200), endoscopists number (≤ 3 and > 3) on summary estimates. Finally, potential publication bias was investigated using Deeks’ funnel plot [26]. We used Stata (version12.1) and MetaDiSc (version1.4) to perform the analyses.

## Results

### Eligible studies

**Fig 1:**
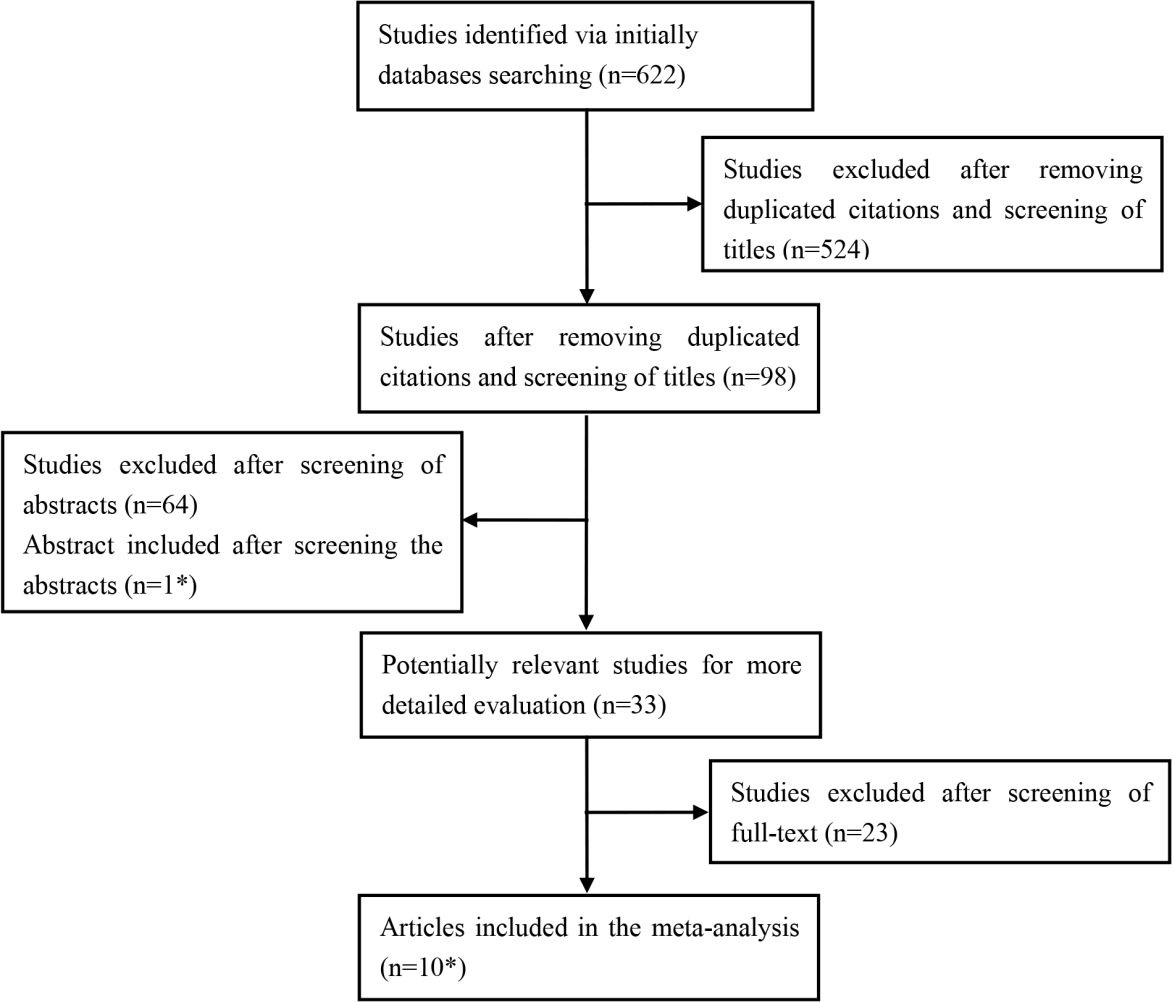
Flow diagram of study selection for the meta-analysis. * Finally, 13 studies were identified from the 10 articles and 1 abstract.

Following the initial keyword search, we got 622 citations in total (Fig 1). We excluded 524 citations that were not associated with i-Scan and colonic polyps after removing duplicated citations and screening the titles. 64 articles that did not focus on the endoscopic diagnosis of colonic polyps with i-Scan were excluded after screening the abstracts. One abstract was included after screening the abstracts. Of the 33 articles left for full text review, 23 articles were excluded as they were retrospective studies (n = 4), studies about detection rate of polyps (n = 6), studies without complete data(n = 2), or for other reasons(n = 11). Eventually, 10 full published [27–36] papers and 1 abstract [37] were selected according to the study inclusion criteria and exclusion criteria. 13 eligible studies were identified from the 11 articles. For two articles, Carlos Robles-Medranda et al. [37] and Sung Noh Hong et al. [35], 2 eligible studies were identified from each of them. These studies from one article were performed with different i-Scan mode and met inclusion criteria.

### Study characteristics

The main characteristics of the included studies were listed in Table 1. The 13 studies [27–37] included a total of 925 patients and 2312 polyps. 51.4% of all polyps were neoplastic verified by histology—the range was from 3.28% to 78.67%. One of the studies did not present information of patients [34]. Three studies [29, 32, 36] were performed in Germany, three [33, 35] in South Korea, two [37] in Ecuador, and each of Italy [31], Netherlands [34], Taiwan [28], UK [30], and USA [27] had one. In 11 studies [27–31, 33, 35–37] the endoscopic diagnosis of polyp was performed in real-time. However, two [32,34] were performed by reading static images which were collected from consecutively enrolled patients. For the diagnostic criteria to predict polyp histology, Kudo pit pattern classification or modified Kudo pit pattern classification was used in six studies [27–29, 32, 35], Narrow Band Imaging International Colorectal Endoscopic (NICE) classification in three [31, 37], and other criteria in four [30, 33, 34, 36]. Six studies [27–28, 30, 33–34, 36] analyzed the small polyps (≤ 10 mm in size). The diminutive polyps (≤ 5 mm in size) were analyzed in two studies [33,36].

**Table 1:** Study characteristics.

Study (year)	Study no.	Country or Area	Study type	Assessment methods	Diagnostic criteria	Endoscopists number	No. of Patients	No. of polyps	No. of neoplasms/No. of non-neoplasms	Mean size of polyps,mm (range or ±SD)
Basford et al (2014)[30]	1	UK	Prospective	Real time	Adapted N.A.C.	1	84	209	134/75	4.2(±2.2)
Bouwens et al (2013)[34]	2	Netherlands	Prospective	Static image	ICE-classification	1 operator 11 raters	N	550	396/154	- (<10)
Carlos et al (2013)*[37]	3	Ecuador	Prospective	Real time	NICE	3	72	122	20/102	-
Carlos et al (2013)*[37]	4	Ecuador	Prospective	Real time	NICE	3	72	122	20/102	-
Chan et al (2012)[27]	5	USA	Prospective	Real time	Kudo	2	43	103	54/49	3.7(2–8)
Han et al (2012) [28]	6	Taiwan	Prospective	Real time	Kudo	5	54	101	57/44	4.2(1–9)
Hoffman et al (2010)[36]	7	Germany	Prospective	Real time	Mucosal pattern and vascular pattern^+^	3	69	335	11/324	- (≤5)
Hoffman et al (2010)[29]	8	Germany	Prospective, randomized	Real time	Kudo and vascular pattern	6	100	145	82/63	5.6(±6.8)
Hong et al (2012)**[35]	9	South Korea	Prospective, randomized	Real time	Modified Kudo and vascular pattern	3	115	116	71/45	-
Hong et al (2012)**[35]	10	South Korea	Prospective, randomized	Real time	Modified Kudo and vascular pattern	3	118	109	74/35	-
Lee et al (2011)[33]	11	South Korea	Prospective	Real time	Mucosal pattern and vascular pattern	1	72	140	74/66	- (≤5)
Neumann et al (2013)[32]	12	Germany	Prospective	Static image	Kudo	4	48	110	77/33	4(2–20)
Pigò et al (2012)[31]	13	Italy	Prospective	Real time	NICE	1	78	150	118/32	6.8(±5.5)

* These two studies were identified from one literature.

** These two studies were identified from one literature.

^+^ This study did not describe their diagnostic criteria in detail.

N.A.C., a previously described classification system developed on the base of characterization of colonic polyps using FICE; ICE-classification, i-Scan classification for endoscopic diagnosis which is a simple classification built upon Kudo classification and NICE classification; NICE, Narrow Band Imaging International Colorectal Endoscopic Classification; N, not mentioned.

### Quality assessment

**Fig 2:**
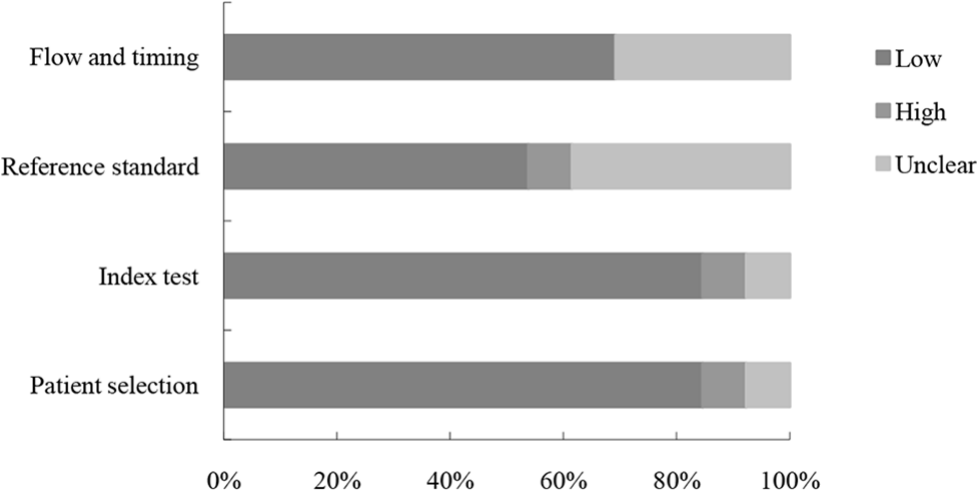
Proportion of studies with low, high, or unclear risk of bias. The vertical axis displays domains of QUADAS-2.

**Fig 3:**
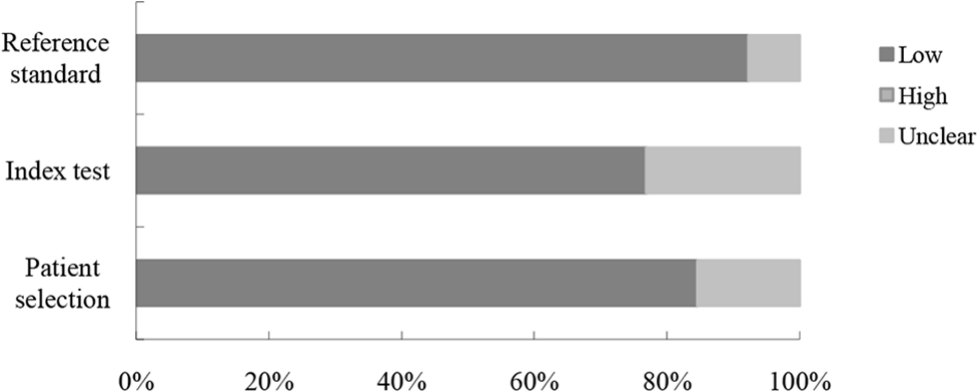
Proportion of studies with low, high, or unclear concerns regarding applicability. The vertical axis displays domains of QUADAS-2.

The quality of the included studies according to the QUADAS-2 tool was summarized and displayed graphically (Figs 2 and 3). In general, the included 13 studies met most of the quality criteria. However, in some studies it was not clear whether histologists were blinded to the endoscopic diagnosis that may induce bias.

### Diagnostic performance of i-Scan diagnosis

**Fig 4:**
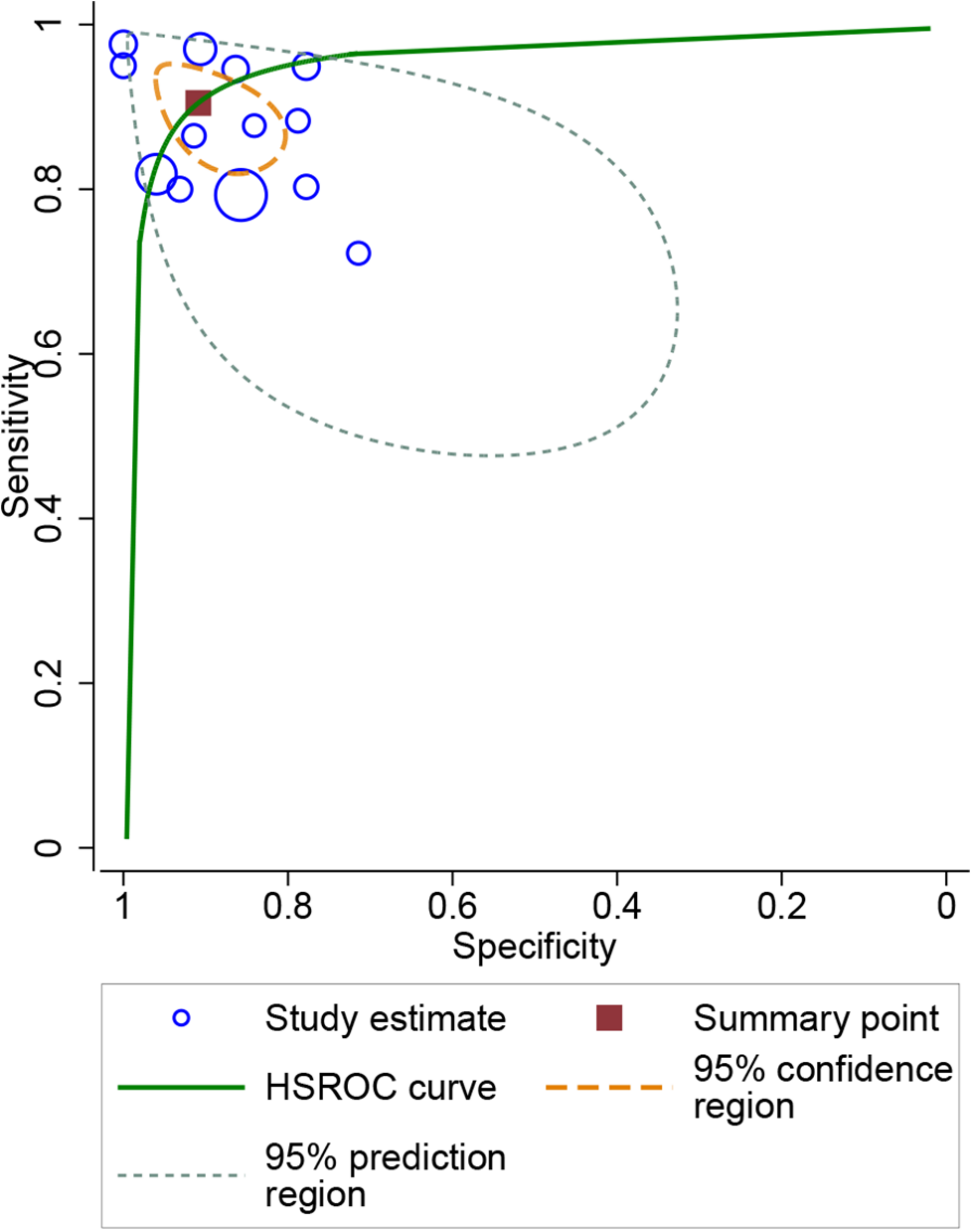
Hierarchical summary receiver-operating characteristic (HSROC) curve for the diagnostic performance of i-Scan. The size of the blue circles indicates the number of polyps in the individual studies. The summary sensitivity and specificity is shown with a dark red square and the 95% confidence region is plotted in short lines. The area under the HSROC curve (AUC) was 0.96 (95%CI 0.94–0.97).

Meta-analysis of all 13 studies showed that the summary sensitivity of i-Scan to predict polyps histology was 90.4% (95%CI 85%-94.1%), and specificity was 90.9% (95%CI 84.3%-94.9%). The summary positive likelihood ratio (LR+) and negative positive likelihood ratio (LR-) was 9.94 (95%CI 5.49–17.98) and 0.10 (95%CI 0.06–0.17), respectively. The area under the HSROC curve (AUC) was 0.96 (95%CI 0.94–0.97) (Fig 4) indicating highly accurate optical diagnostic performance of i-Scan.

**Fig 5:**
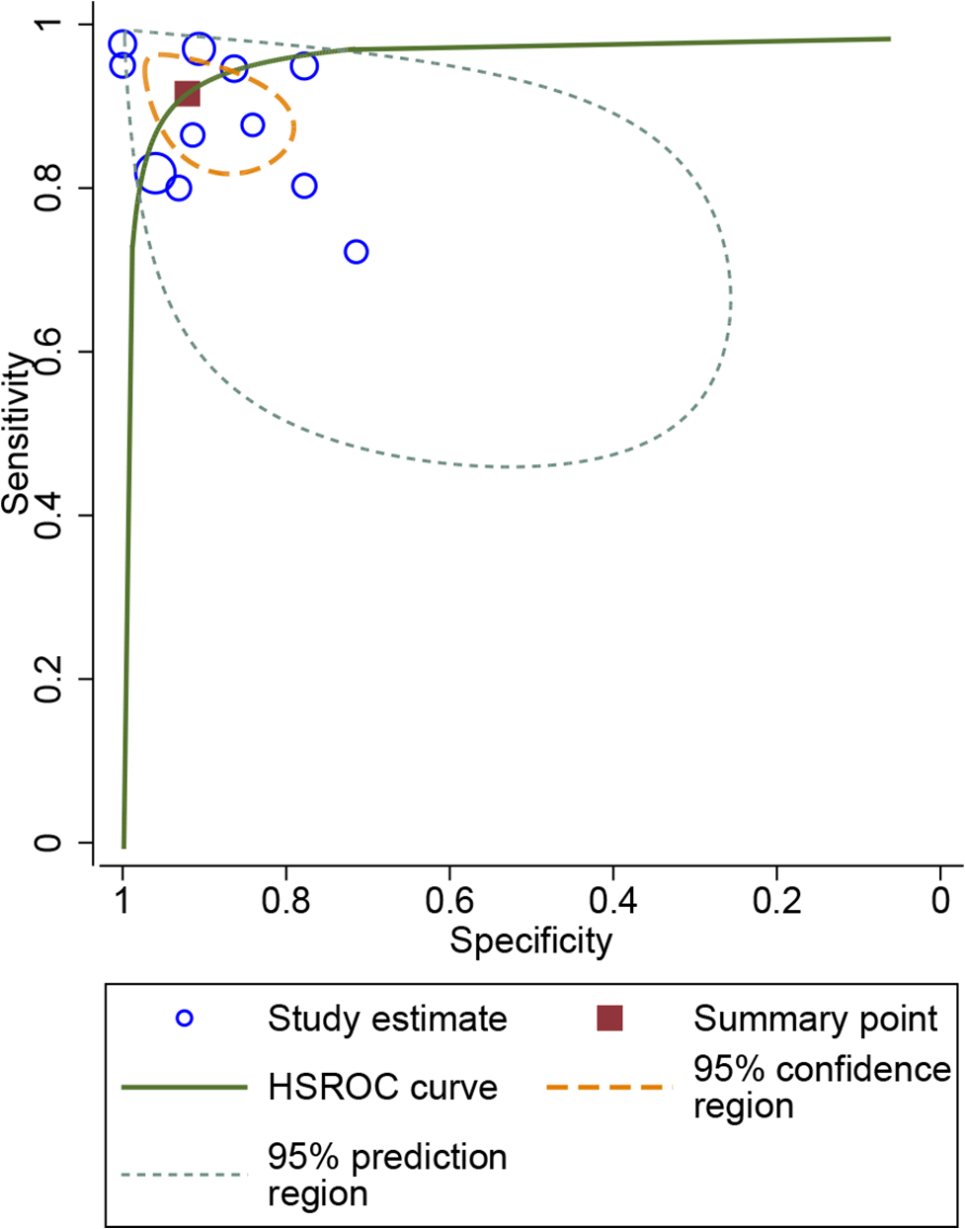
Hierarchical summary receiver-operating characteristic (HSROC) curve for the diagnostic performance of i-Scan to predict colonic polyps histology in real-time. The size of the blue circles indicates the number of polyps in the individual studies. The summary sensitivity and specificity is shown with a dark red square and the 95% confidence region is plotted in short lines. The AUC was 0.97 (95%CI 0.95–0.98).

We principally performed subgroup analysis for studies with histology prediction of polyps in real-time, and studies using different criteria. The subgroup of histology prediction in real-time composed of 11 studies enrolling 1652 polyps. In the 11 studies, the summary sensitivity and specificity was 91.5% (95%CI 85.7%-95.1%) and 92.1% (95%CI 84.5%-96.1%), respectively. The LR+ and LR- was 11.6 (95%CI 5.61–23.81) and 0.09 (95%CI 0.05–0.16), respectively. The AUC was 0.97 (95%CI 0.95–0.98) (Fig 5) indicating a high accuracy of i-Scan to differentiate neoplastic and non-neoplastic polyps in real-time. In the 6 studies (684 polyps) in which histology prediction was performed with Kudo or modified Kudo classification, sensitivity and specificity was 86.3% (95%CI 82.7%-89.5%) and 84.8% (95%CI 79.9%-88.8%), respectively. For the 3 studies (394 polyps) using NICE, the sensitivity and specificity was 93.0% (95%CI 87.9%-96.5%) and 94.4% (95%CI 90.6%-97.0%), respectively. In 4 studies (1234 polyps) with other criteria, the sensitivity and specificity was 85.0% (95%CI 82.0%-87.8%) and 91.8% (95%CI 89.3%-93.8%), respectively. For these three groups with different criteria, of which the data were not plotted in a HSROC curve, we just calculated summary estimates of them. For the sub-group analysis of small polyps (≤ 10 mm) in six studies (1438 polyps), sensitivity and specificity was 89.3% (95%CI 79.5%-94.7%) and 88.3% (95%CI 80.7%-93.2%), respectively, with AUC of 0.95(0.92–0.96). The sensitivity and specificity of diagnosis of diminutive polyps (≤ 5 mm) was 92.9% (95%CI 85.3%-97.4%) and 94.4% (95%CI 91.6%-96.4%), respectively. The main results were shown in Table 2.

**Table 2:** Accuracy of i-Scan for optical diagnosis of colonic polyps.

Study group	No.of studies(no. of polyps)	Summary estimates (95%CI)		Likelihood ratio (95%CI)		Area under HSROC curve (95%CI)
		Sens	Spec	LR+	LR-	
ALL	13(2312)	90.4 (85.0–94.1)	90.9 (84.3–94.9)	9.94 (5.49–17.98)	0.10 (0.06–0.17)	0.96 (0.94–0.97)
Real-time	11(1652)	91.5 (85.7–95.1)	92.1 (84.5–96.1)	11.6 (5.61–23.81)	0.09 (0.05–0.16)	0.97 (0.95–0.98)
Criteria						
Kudo	6(684)	86.3 (82.7–89.5)	84.8 (79.9–88.8)	5.00 (2.69–9.30)	0.17 (0.10–0.30)	*
NICE	3(394)	93.0 (87.9–96.5)	94.4 (90.6–97.0)	11.59 (3.18–42.28)	0.11 (0.05–0.25)	*
Others	4(1234)	85.0 (82.0–87.8)	91.8 (89.3–93.8)	9.31 (4.97–17.45)	0.10 (0.03–0.35)	*
Size						
Polyps≤10mm	6(1438)	89.3(79.5–94.7)	88.3(80.7–93.2)	7.62(4.31–13.5)	0.12(0.06–0.25)	0.95(0.92–0.96)
Polyps≤5mm	2(475)	92.9(85.3–97.4)	94.4(91.6–96.4)	11.9(3.20–44.29)	0.10(0.03–0.30)	*

* These four groups were not plotted in a HSROC curve.

### Test for heterogeneity

The heterogeneity, however, was presented in the overall studies with Q of 5.001 (Chi-square, p = 0.041) and I^2^ (I-square) of 60% indicating a high heterogeneity in the overall studies. There was no threshold effect inducing heterogeneity (Spearman’s coefficient: -0.322, p 0.284). Then we performed subgroup analyses to find the source of heterogeneity to assess the effect of assessment methods (real-time or not), diagnostic criteria (Kudo, NICE and others), and polyps size (polyps ≤ 10 mm or polyps ≤ 5 mm) on summary estimates. The mode of i-Scan was varied in most studies, so we did not analyze it. This may be one of the sources of heterogeneity. For the subgroup of real-time, the I^2^ (I-square) was 44% indicating a moderate heterogeneity. For the subgroup of small polyps (≤ 10 mm), the I^2^ (I-square) was 25% indicating a mild heterogeneity.

### Publication bias estimate

**Fig 6:**
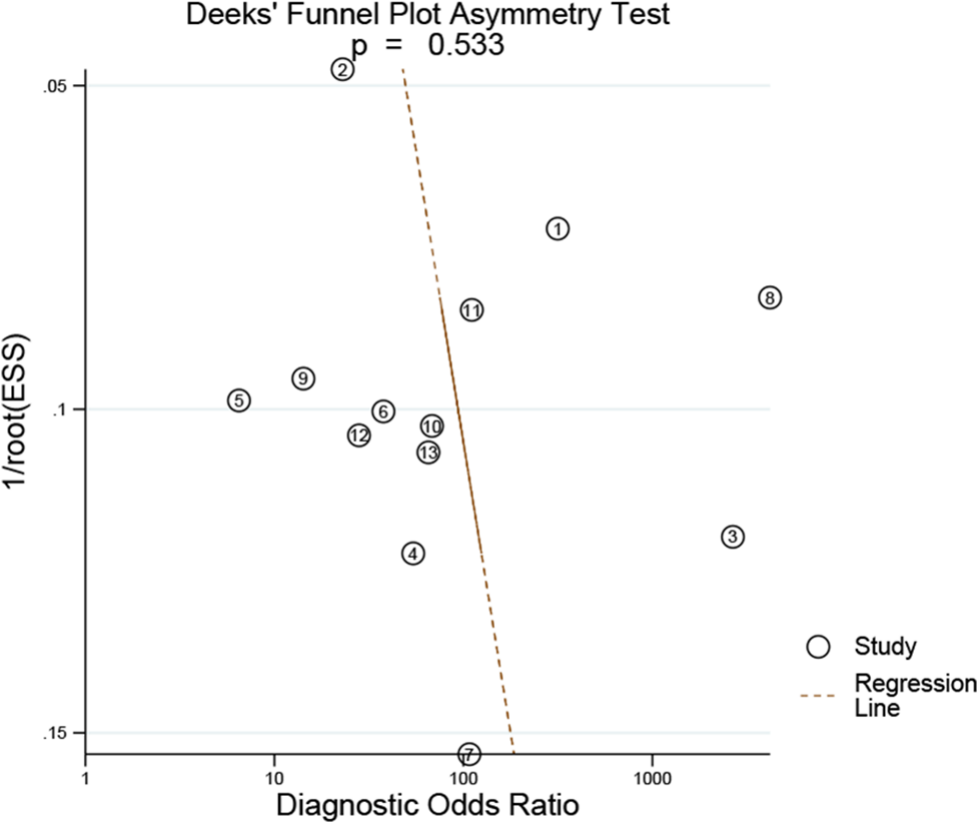
Deeks’ funnel plot to evaluate publication bias. The vertical axis displays the inverse of the square root of the effective sample size (1/root(ESS)). The horizontal axis displays the diagnostic odds ratio (DOR). P = 0.533 indicates a symmetrical funnel shape and suggests that publication bias is absent.

We used Deeks’ funnel plot [26] to assess the potential publication bias of the overall studies. A slope coefficient of 12.8 (p = 0.533) in the Deeks’ funnel plot (Fig 6) asymmetry test indicates a symmetrical funnel shape and suggests that publication bias is absent.

## Discussion

This meta-analysis summarized the available evidence regarding the accuracy of i-Scan for optical diagnosis of colonic polyps. The overall results of this meta-analysis indicated that i-Scan had accurate optical diagnosis performance, with an area under the hierarchical summary receiver-operating characteristic (HSROC) curve of 0.96. For i-Scan predicting polyps histology in real-time, it also showed a high accuracy with an area under the HSROC curve of 0.97. Endoscopy with i-Scan correctly diagnosed 91.5% of neoplasms and 92.1% of non-neoplastic polyps in real-time. Comparing with narrow band imaging, i-Scan has a similar sensitivity (91.5% vs 91%) and a higher specificity (92.1% vs 82.6%) to differentiate neoplastic from non-neoplastic colonic polyps in real-time according to a meta-analysis of Sarah K McGill et al. [38]. Similarly, comparing with fujinon intelligent color enhancement (FICE), i-Scan has a similar sensitivity (91.5% vs 91.8%) and a higher specificity (92.1% vs 83.5%) to differentiate neoplastic from non-neoplastic colonic polyps in real-time according to a meta-analysis of Linda K Wanders et al. [39]. Our findings may be explained by the fact that i-Scan is generally integrated with high-definition colonoscopy, and high-definition colonoscopy could have better performance than standard definition colonoscopy in detecting polyps [40]. However, some studies included in the above two meta-analyses were based on standard definition colonoscopies [41–45].

The criteria adopted for predicting colonic polyps histology when performing optical diagnosis of colonic polyps with i-Scan varied in the included studies. As to subgroups with different diagnostic criteria, Narrow Band Imaging International Colorectal Endoscopic (NICE) classification showed higher sensitivity and specificity (93% and 94.4% respectively) than the other two criteria. However, we were not sure if the difference was statistically significant, because of the overlapped 95% confidence interval. Only three studies with NICE as criteria were included. NICE had shown high accurate diagnosis of colonic lesions, whereas it was designed on the basis of NBI characters [21,22]. So more studies need to be done to make sure if it is suitable to i-Scan. More studies included in the meta-analysis adopted Kudo pit pattern classification or modified Kudo pit pattern classification as their diagnostic criteria showing sensitivity of 86.3% and specificity of 84.8%. Kudo pit pattern classification, however, was designed only on the pit pattern of colonic lesions [19,20]. One included studied adopted N.A.C. as the diagnostic criteria, which was a classification system developed on the basis of characterization of colonic polyps using FICE [46]. Both of these criteria are not specialized for i-Scan. While only three studies [33, 34, 36] were performed using their own criteria adjusted to the characters of i-Scan. To internationally standardize the i-Scan observation criteria, a simple effective classification system is required. Further studies validating a specific polyp classification system specialized for i-Scan may be necessary. In this way, i-Scan will be more widely applied in clinical practice.

The size of polyps is always related to pathological grade and endoscopy accuracy and diminutive colon polyps (≤ 5 mm) rarely have malignant potential[7]. In our study, i-Scan showed accurate optical diagnostic performance for optical diagnosis of small and diminutive polyps. For the sub-group analysis of small polyps (≤ 10 mm), sensitivity and specificity of i-Scan was 89.3% and 88.3%, respectively. The sensitivity and specificity for diminutive polyps (≤ 5 mm) was 92.9% and 94.4%, respectively. i-Scan seemed have better diagnostic performance for diminutive polyps numerically. However, we were not sure if the difference was statistically significant, because of the overlapped 95% confidence interval.

i-Scan could combine 6 digital chromoendoscopic post-processing settings (v, p, e, b, g and c) called tone enhancement (TE) with different levels of contrast enhancement (CE) and surface enhancement (SE) resulting in diverse combinations [17]. TE is designed to enhance minute surface structures and subtle changes in color and evaluate the lesions in detail. SE and CE can improve identification of lesions without markedly reducing the brightness of images and altering the color tone. Each mode can be used along or combined with other modes to get operators preferred images. There are 3 established modes combinations (i-Scan 1, 2 and 3) currently available presented in the instrument. The combination of multiple modes can provide operator preferred images. We are not sure which one is the most suitable combination of modes for optical diagnosis of colonic polyps. Carlos Robles-Medranda et al. [37] established 3 new i-Scan setting (NIS) modes measuring their effectiveness for the real-time histological prediction of colonic polyps and found that their NIS modes were effective for histological prediction of colonic polyps in real-time. More studies need to be done to establish a general accepted setting specifically for histological prediction of colonic polyps.

In our study, we analyzed accuracy of i-Scan in the diagnosis of colonic polyps in real-time. This is the first meta-analysis assessing the performance of different diagnostic criteria for predicting colonic polyps histology with i-Scan. We performed the analyses using a bivariate meta-analysis following a random effects model to calculate overall estimates of sensitivity and specificity，allowing more intra- and inter-study variability than a fixed-effect model [47]. This model allows researchers to avoid misleading conclusions.

Nonetheless, the main limitation to our study is that various i-Scan modes and different diagnostic criteria were adopted when performing optical diagnosis with i-Scan. The non-uniform diagnostic criteria may restrict the application of i-Scan. Though we performed subgroup analyses, such as real time, diagnostic criteria, polyp size, it is not comprehensive. In most studies, it is not clear whether the endoscopic diagnoses of polyps were performed with high confidence. Not all of the included studies provided the information of polyps location, polyps morphology and proportion of exact pathological type. The incomplete information restricts further analyses. The relatively high heterogeneity presented across the 13 included studies is also the limitation of this study. Though the heterogeneity was reduced in subgroup analyses, moderate heterogeneity was still present in some subgroup analyses. The relative percentage of neoplasia to non-neoplastic fluctuates between 78.67% and 3.28% indicating non-uniformity of all studies. The discrepancy in the included studies may be caused by diverse target population, such as American, Asian and European, and the population composition of the individual studies. For 1 article and 1 abstract in the study, two studies were identified from each of them. The two studies from one literature may have potential impropriety, though there is no obvious change in the results as the analysis being repeated after removing one of them. The above situation may induce heterogeneity.

## Conclusions

Endoscopic diagnosis with i-Scan is an accurate optical diagnosis technique to differentiate neoplastic from non-neoplastic polyps, with an area under the hierarchical summary receiver-operating characteristic (HSROC) curve exceeding 0.90. Both the sensitivity and specificity for diagnosing colonic polyps are over 90%.

## Supporting Information

S1 Table PRISMA checklist. The meta-analysis was performed in accordance with the Preferred Reporting Items for Systematic reviews and Meta-Analyses (PRISMA) guidelines. Every section of the meta-analysis is noted in the PRISMA checklist.
(DOC)
